# Urinary incontinence as a main clinical manifestation of early stage Wernicke’s encephalopathy: a case report

**DOI:** 10.3389/fneur.2023.1157806

**Published:** 2023-05-24

**Authors:** Haitian He, Kebing Wang, Yang Wang, Jinbin Luo

**Affiliations:** ^1^Department of Urology, Shenzhen Qianhai Shekou Free Trade Zone Hospital, Shenzhen, China; ^2^Department of Urology, Huazhong University of Science and Technology Union Shenzhen Hospital, Shenzhen, China

**Keywords:** Wernicke encephalopathy, female patient, intestinal obstruction, urinary incontinence, vitamin B1

## Abstract

Wernicke’s encephalopathy (WE) is a condition caused by a deficiency of vitamin B1. While there have been many reported cases of WE in the literature, there are few reports on the early stages of the disorder. In this report, we present a case of WE with urinary incontinence as the main clinical manifestation. A 62-year-old female patient was admitted to the hospital due to intestinal obstruction and did not receive vitamin B1 supplements for 10 days. Three days after her operation, she developed urinary incontinence. She also had mild mental symptoms, such as a little indifference. After consultation with a urologist and neurologist, the patient was immediately given intramuscular vitamin B1 at a dosage of 200 mg/day. After 3 days of supplementing with vitamin B1, her urinary incontinence and mental symptoms improved and were completely resolved after 7 days of treatment. Surgeons should be aware that when long-term fasting patients have urinary incontinence, it may be a symptom of WE, and they should be supplied with vitamin B1 in a timely manner without extensive examination.

## Introduction

Wernicke’s encephalopathy (WE), first reported by Carl Wernicke in 1881, is a condition caused by a deficiency of vitamin B1 (1). Common causes of WE include chronic alcoholism, gastrointestinal diseases, gastrointestinal surgery, and severe vomiting during pregnancy. WE can be difficult to diagnose due to its varied presentation, but typical symptoms include ataxia, nystagmus, ophthalmoplegia, confusion, and these symptoms are rarely seen together during early stages (2). WE can also cause urinary incontinence and other micturitional disorders (3). Many urologists are not familiar with WE, which is why we are reporting a case of WE with urinary incontinence as the main manifestation.

## Case report

A 62-year-old female patient was admitted to our hospital with complaints of intestinal obstruction, nausea, and vomiting for 5 days. Prior to her illness, she was in good physical condition and had no history of alcoholism, liver disease, digestive disorders, or any surgeries. A CT scan of the abdomen revealed a mass in the right colon, which was resected and a proximal colostomy was performed. The surgery had no complications. The patient received continuous intravenous nutrition, but did not receive vitamin B1. After the surgery, the color Doppler ultrasound examination showed that there was no remaining urine in the bladder. Three days after the surgery with general anesthesia, she developed total urinary incontinence without any obvious cause. She had no confusion, could answer questions normally, but had mild indifference in her mental state manifested as not responding promptly when called by name and showing no interest in changes in the surrounding people or environment, no ataxia, no nystagmus and ophthalmoplegia, and her Mini-Mental State Exam (MMSE) score was 27 with difficulty concentrating, daydreaming, making errors in simple calculations, and being unable to recall events that occurred 10 min ago. Physical examination revealed a temperature of 37.6°C, heart rate of 85 per minute, respiration of 18 per minute, and blood pressure of 135/82 mmHg. She was cooperative during examination, pupils were 2.5 mm, equally reactive to light, no obvious ataxia, no nystagmus and ophthalmoplegia, cardiopulmonary examination was normal, had slight abnormal involuntary movements in the extremities, Babinski reflex and Kernig sign were negative. Laboratory test results were normal, except for slightly lower albumin levels.

The general surgeon was unable to determine the cause of the patient’s urinary incontinence, so they consulted with a urologist. After the consultation, the urologist considered the possibility that injury to the lumbar or sacral nerves may have caused the incontinence. However, the general surgeon denied the possibility of nerve damage. The neurologist did not consider the possibility of cerebral hemorrhage, cerebral thrombosis, or other central neuropathies as the patient had no previous history of neurological disease. Therefore, a diagnostic supplement of vitamin B1 was recommended, and the patient was given intramuscular vitamin B1 immediately at a dose of 200 mg/day.

After 3 days of supplementing with vitamin B1, the patient’s urinary incontinence and mental indifference improved. The patient’s urinary incontinence completely resolved within 7 days of treatment.

## Discussion

WE is caused by a deficiency of vitamin B1. Vitamin B1 is an important coenzyme in the tricarboxylic acid cycle and cannot be synthesized by the body, and it also has no storage in our bodies. In our case report, before experiencing the obvious symptoms of intestinal obstruction, nausea and vomiting, the patient had already shown signs of bloating, poor appetite, and reduced food intake for about 10 days. After admitted to our hospital for 3 days, the patient received the surgery. Three days after the surgery, she developed urinary incontinence without any obvious cause. The long-term lack of nutrition and the long-term fasting may result in vitamin B1 deficiency. If the body is unable to take in vitamin B1, deficiency can occur quickly. Its deficiency can cause disorders in glucose metabolism, insufficient energy production in cells, and accumulation of pyruvic acid and lactic acid in cells, which can all lead to changes in nerve and brain tissue function and result in corresponding symptoms and signs (1, 4).

WE is difficult to diagnose due to its varied presentation. The typical symptoms of WE are ataxia, nystagmus, ophthalmoplegia, and confusion, although they are rarely seen together (2). Laboratory tests are not able to directly diagnose WE, as there are no specific abnormalities detectable in cerebrospinal fluid, EEG, or evoked potentials (5). MRI is the most valuable method for confirming a diagnosis of WE. It shows symmetric involvement of the mammillary bodies, the tectal plate, the periaqueductal grey matter and the periventricular region of the third ventricle, including the paramedian thalamic nuclei. Signal hyperintensities on T2-weighted sequences, FLAIR, and diffusion-weighted images within the posteromedial thalami and surrounding the third ventricle are the most common abnormality seen in patients with WE (6).

As is well known, normal urinary function is regulated by the central nervous system. Lower urinary tract afferents synapse in the dorsal horn of the spinal cord and ascend to the midbrain periaqueductal gray (PAG) (7). In the brainstem, the pontine micturition center (PMC) is a convergence point for multiple influences and serves as a coordinating center for voiding (7). The influence of these centers on the PMC is primarily mediated via the periaqueductal gray, which also integrates bladder sensory information, modulates voiding and storage of urine, and controls the transition between the two phases (7, 8). Nerve and brain tissues are mainly powered by glucose. When there is a deficiency of vitamin B1, it causes disorders in glucose metabolism and insufficient energy production, at the same time, it can cause the accumulation of pyruvic acid and lactic acid in nerve cells of PAG, leading to voiding dysfunction or incontinence.

The main treatment for WE is the intravenous or intramuscular injection of Vitamin B1. The most recent European Federation of the Neurological Societies (EFNS) guideline for the therapy of WE recommend: (1) Vitamin B1 use in suspected or confirmed patients. (2) A dose of Vitamin B1 of 200 mg, three times daily. (3) Supplementation before carbohydrate consumption, followed by a normal diet. (4) Treatment should continue until clinical symptoms and signs can no longer be improved (9). The dosage of Vitamin B1 used to treat WE varies among studies, and there is currently no uniform dosage. Some researchers suggest that the dosage should be tailored to the patient’s condition, such as weight and time of fasting (10). In our case, the patient had not eaten anything for 5 days and had not received Vitamin B1 supplementation for 5 days after being admitted to the hospital. We administered Vitamin B1 intramuscularly at a dose of 200 mg/day.

There have been many reports of WE in the literature, however, there are few reports on the early stages of WE with urinary incontinence as the main clinical manifestation. Kühn et al., reported a 40-year-old woman who underwent gastroplasty combined with gastric banding for severe obesity. After 3 months, she developed convergent strabismus, apathy, and urinary incontinence, which was diagnosed as WE and treated accordingly. Even after 6 months, her recovery remained incomplete as she still showed gait difficulties and nystagmus (11). This patient had the similar symptoms of urinary incontinence as the case reported in our study, and vitamin B1 supplementation was found to have improved these symptoms. Yaguchi et al. reported a case of a 54-year-old man with alcoholic WE who immediately regained consciousness after the administration of thiamine. Although urinary retention persisted, a urodynamic study revealed detrusor-sphincter dyssynergia (3). The study suggested that lesions of the periaqueductal gray matter and/or the dorsolateral portion of the pons were responsible for the micturitional disturbance in the patient (3). The symptoms of this patient were completely opposite to those reported in the first case, indicating that damage to different brain tissues could lead to entirely different clinical symptoms. Thus, the clinical manifestations of WE are diverse.

WE is not frequently encountered in clinical settings, and as a result, urologists are not well-versed in its diagnosis and treatment. The symptoms of WE can vary greatly, but typically involve issues such as eye muscle paralysis, lack of coordination, and cognitive and/or consciousness-related problems. Instances of WE characterized by urinary incontinence as the primary symptom are infrequent. In fact, we have only encountered one such case in our clinical experience thus far, which implies that this study has certain constraints. To bolster the evidence, it may be worthwhile to evaluate the thiamine levels in whole blood. In light of these findings, further research is warranted to better understand the underlying causes of WE, and additional clinical cases should be examined. Urinary incontinence can commonly occur as a result of spinal anesthesia (12), while general anesthesia was used in our case report. Damage to pelvic nerves or muscles and injury to the bladder or urethra can be common causes for total incontinence after abdominal surgery (13, 14). Although we have not observed any surgical damage to these tissues in this patient, we should be cautious when interpret our findings, as these possibilities can not be completely ruled out. We suggest that when patients who have been fasting for a long time and not receiving vitamin B1 supplements develop symptoms such as mental indifference, urinary incontinence, and fecal incontinence, the possibility of WE should be considered. Diagnostic vitamin B1 supplementation can be given to patients without the need for cerebral MRI, cerebrospinal fluid examination, and other examinations.

## Data availability statement

The original contributions presented in the study are included in the article/Supplementary material, further inquiries can be directed to the corresponding author.

## Ethics statement

The studies involving human participants were reviewed and approved by the Ethics Committee of Shenzhen Qianhai Shekou Free Trade Zone Hospital. The patients/participants provided their written informed consent to participate in this study. Written informed consent was obtained from the participant/patient(s) for the publication of this case report.

## Author contributions

HH and KW performed the data collection and wrote the manuscript. YW and JL analyzed the data. All authors contributed to the article and approved the submitted version.

## Funding

This research was funded by the Natural Science Foundation of Guangdong Province (2020A1515010202), Technology and Innovation Commission of Shenzhen Municipality (JCYJ20180302144556243).

## Conflict of interest

The authors declare that the research was conducted in the absence of any commercial or financial relationships that could be construed as a potential conflict of interest.

## Publisher’s note

All claims expressed in this article are solely those of the authors and do not necessarily represent those of their affiliated organizations, or those of the publisher, the editors and the reviewers. Any product that may be evaluated in this article, or claim that may be made by its manufacturer, is not guaranteed or endorsed by the publisher.
